# Outcomes of patients with secondary central nervous system lymphoma following CAR T-cell therapy: a multicenter cohort study

**DOI:** 10.1186/s13045-023-01508-3

**Published:** 2023-11-09

**Authors:** Narendranath Epperla, Lei Feng, Nirav N. Shah, Lindsey Fitzgerald, Harsh Shah, Deborah M. Stephens, Catherine J. Lee, Thomas Ollila, Geoffrey Shouse, Alexey V. Danilov, Kevin A. David, Pallawi Torka, Hamza Hashmi, Brian Hess, Stefan K. Barta, Jason T. Romancik, Jonathon B. Cohen, Kaitlin Annunzio, Adam S. Kittai, John Reneau, Joanna Zurko, Imran A. Nizamuddin, Jane N. Winter, Leo I. Gordon, Shuo Ma, Romil Patel, Loretta Nastoupil, Sairah Ahmed, Reem Karmali

**Affiliations:** 1 Division of Hematology, Department of Medicine, Arthur G. James Cancer Hospital and Richard J. Solove Research Institute, The Ohio State University, Columbus, OH 43210 USA; 2 University of Texas MD Anderson Cancer Center, Houston, TX USA; 3 Medical College of Wisconsin, Milwaukee, WI USA; 4 Huntsman Cancer Institute, University of Utah, Salt Lake City, UT USA; 5 Fred Hutchinson Cancer Center, University of Washington Medical Center, Seattle, WA USA; 6 Lifespan Cancer Institute, Brown University, Providence, RI USA; 7 City of Hope National Medical Center, Duarte, CA USA; 8 Rutgers Cancer Institute of New Jersey, Rutgers University, New Brunswick, NJ USA; 9 Memorial Sloan Kettering Cancer Center, New York, NY USA; 10 Roswell Park Comprehensive Cancer Center, Buffalo, NY USA; 11 Hollings Cancer Center, Medical University of South Carolina, Charleston, SC USA; 12 Abramson Cancer Center, University of Pennsylvania, Philadelphia, PA USA; 13 Winship Cancer Institute, Emory University, Atlanta, GA USA; 14 Robert H Lurie Comprehensive Cancer Center, Northwestern University, Chicago, IL USA

**Keywords:** CAR-T, Secondary CNS lymphoma, SCNSL, Outcomes, PFS, OS

## Abstract

Chimeric antigen receptor T-cell therapy (CAR-T) has been successful in treating relapsed/refractory B-cell lymphomas. However, its role in the treatment of diseases involving the central nervous system (CNS) is not well studied. We performed a multicenter retrospective cohort study to evaluate the outcomes of patients with secondary CNS lymphoma (SCNSL) who received CAR-T. Eligibility required active CNSL at the time of apheresis. The objectives included evaluation of overall survival (OS), progression-free survival (PFS), identification of predictors of complete response (CR) post-CAR-T, and assessment of risk factors for cytokine release syndrome (CRS) and immune effector cell-associated neurotoxicity syndrome (ICANS). Sixty-one patients were included in the analysis. The overall response rate was 68% with a CR rate of 57%. In the multivariable analysis, patients who experienced any grade CRS had higher odds of achieving CR (OR = 3.9, 95% CI = 1.01–15.39, *p* = 0.047). The median PFS was 3.3 months (95% CI = 2.6–6.0 months) with 6- and 12-month PFS rates of 35% and 16%, respectively. The median OS was 7.6 months (95% CI = 5.0–13.5 months) with 6- and 12-month OS rates of 59% and 41%, respectively. Any grade CRS and ICANS were 70% (n = 43) and 57% (n = 34), respectively with grade ≥ 3 CRS and ICANS rates of 16% and 44%. Factors associated with increased risk of CRS and ICANS included receiving axi-cel or having leptomeningeal ± parenchymal + CNS involvement, respectively. Despite achieving high response rates, most patients experience early relapse or death following CAR-T in SCNSL. The current study provides a benchmark for future trials exploring novel therapeutic options in SCNSL.

**Supplementary Information:**

The online version contains supplementary material available at 10.1186/s13045-023-01508-3.

To the Editor,

The involvement of the central nervous system (CNS) by recurrent large B-cell lymphoma (LBCL) may be the result of either secondary CNS dissemination from systemic lymphoma—secondary CNS lymphoma (SCNSL) or relapsed primary CNS lymphoma (PCNSL). The prognosis remains dismal in either case with no consensus regarding optimal salvage treatment [1]. While whole brain radiotherapy (WBRT) can improve survival by 10–16 months in recurrent PCNSL, it is associated with significant cognitive effects [2–4]. Different therapeutic strategies have been tried with modest success; however, outcomes of patients with SCNSL remain poor.

Chimeric antigen receptor T-cell therapy (CAR-T) has revolutionized the management of patients with relapsed or refractory (R/R) LBCL in the third line [5–7] as well as the second line setting [8, 9] including patients who are transplant ineligible with FDA approval in both these settings. However, the majority of pivotal CAR-T clinical trials excluded patients with CNS involvement. Limited small retrospective and prospective studies have evaluated CAR-T in patients with CNS involvement with LBCL (Additional file 1: Table S1 outlines the studies with n ≥ 5). Herein, we sought to evaluate the outcomes of patients with SCNSL who received CAR-T using a multicenter retrospective cohort study.

This multicenter retrospective cohort study included adult patients (18 years or older) who received CAR T-cell therapy for SCNSL on or after Jan 1, 2018, at 10 US medical centers. The patients must have had active CNS lymphoma at the time of apheresis to be eligible for the analysis. Patients who received CAR-T for R/R primary CNS lymphoma were not included in the current study. The study was approved by the institutional review boards at all participating sites and was conducted in compliance with the Declaration of Helsinki.

The primary objective was to evaluate overall survival (OS). Secondary objectives included the evaluation of progression-free survival (PFS), identification of predictors of complete response (CR) post CAR-T, and assessment of risk factors for cytokine release syndrome (CRS) and neurotoxicity (NT). OS was defined as the date of CAR-T infusion to the date of death or last follow-up. Patients who were alive were censored at the last follow-up date. PFS was defined as the date of CAR-T infusion to date of progression or death, whichever happened first. Patients who were alive without progression were censored at the last follow-up date. Assessment of toxicity and response and statistical analysis are detailed in the Additional file 1.

A total of 61 patients met the eligibility criteria and were included in the analysis. Table 1 shows the key baseline characteristics of the patients stratified by the site of relapse (CNS versus CNS and systemic) prior to CAR-T. The median age at diagnosis was 56 years (range, 18–82 years) and 56% were male. The most common histology was de novo DLBCL (n = 48, 79%) followed by transformed lymphoma (n = 7, 11%). Sixteen patients (30%) had High-grade B-cell lymphoma with *MYC/BCL2* and/or *BCL6* rearrangements (double hit lymphoma [DHL]). Thirty-five patients (69%) had an IPI of 3 or higher at diagnosis. 67% (n = 41) of patients had systemic involvement at diagnosis, while 33% (n = 20) had systemic and CNS involvement. During the first-line (induction) therapy, 24 patients received high-dose methotrexate with systemic therapy (either intercalated or after completion of the systemic therapy).

**Table 1:** Patient Characteristics stratified based on the site of relapse—CNS only relapse versus CNS and systemic relapse prior to CAR-T

Variable	All patients N = 61 (%)	CNS only relapse N = 20 (%)	CNS and systemic relapse N = 41 (%)	p-value
Patient related				
Age at diagnosis				0.79
Median, range (yrs)	56 (18–82)	56 (31–82)	56 (18–73)	
> 60 yrs	17 (18)	6 (30)	11 (27)	
≤ 60 yrs	44 (72)	14 (70)	30 (73)	
Sex, n (%)				0.31
Males	34 (56)	13 (65)	21 (51)	
Females	27 (44)	7 (35)	20 (49)	
COPD				1.00
Yes	3 (5)	1 (5)	2 (5)	
No	58 (95)	19 (95)	39 (95)	
Cardiac co-morbidities*				0.73
Yes	12 (20)	3 (15)	9 (22)	
No	49 (80)	17 (85)	32 (78)	
Disease related				
Histologic subtype				0.22
De novo DLBCL	50 (82)	16 (80)	34 (83)	
tFL	5 (8)	1 (5)	4 (10)	
tMZL	2 (3)	2 (10)	0 (0)	
Others**	4 (7)	1 (5)	3 (7)	
DHL	16 (30)	0 (0)	16 (43)	**0.003**
IPI at diagnosis				0.67
0	1 (2)	0 (0)	1 (3)	
1–2	15 (29)	6 (37)	9 (26)	
≥ 3	35 (69)	10 (63)	25 (71)	
Primary refractory disease^				0.34
Yes	15 (25)	3 (15)	12 (30)	
No	45 (75)	17 (85)	28 (70)	
CAR-T related				
Lines of therapy prior to CART				0.21
Median, range	3 (1–5)	3 (2–5)	3 (1–5)	
≤ 2	21 (36)	9 (45)	12 (31)	
3	27 (46)	6 (30)	21 (54)	
≥ 4	11 (19)	5 (25)	6 (15)	
Prior HD-MTX containing salvage regimen				0.52
Yes	24 (41)	7 (35)	17 (44)	
No	35 (59)	13 (65)	22 (56)	
Prior auto-HCT				**0.03**
Yes	14 (23)	8 (40)	6 (15)	
No	47 (77)	12 (60)	35 (85)	
Median time from diagnosis to CAR-T, range (months)	15.8 (3.1–257.4)	23.1 (3.6–101.1)	13.3 (3.1–257.4)	**0.04**
CAR-T product				0.54
Axi-cel	30 (49)	9 (45)	21 (51)	
Tisa-cel	19 (31)	7 (35)	12 (29)	
Liso-cel	11 (18)	3 (15)	8 (20)	
Brexu-cel	1 (2)	1 (5)	0	
Median age at apheresis, range (yrs)	58 (18–83)	59 (53–83)	58 (46–74)	0.39
ECOG PS at apheresis				**0.02**
0–1	47 (77)	19 (95)	28 (68)	
≥ 2	14 (23)	1 (5)	13 (32)	
Median serum creatinine at apheresis, range	0.87 (0.40–1.67)	0.85 (0.49–1.67)	0.87 (0.40–1.20)	0.92
Site of CNS involvement				**0.04**
Parenchymal	25 (42)	13 (65)	12 (30)	
Leptomeningeal	29 (48)	6 (30)	23 (58)	
Both	6 (10)	1 (5)	5 (12)	
Best response to last line of therapy prior to CAR-T				** < 0.001**
CR	3 (5)	2 (10)	1 (2)	
PR	19 (31)	10 (50)	9 (22)	
Chemorefractory	39 (64)	8 (40)	31 (76)	
Bridging				0.56
Chemo only	23 (38)	8 (40)	15 (37)	
Chemo + XRT	5 (8)	2 (10	3 (7)	
XRT only	9 (15)	1 (5)	8 (19)	
None	24 (39)	9 (45)	15 (37)	
Best response to CAR-T among evaluable patients (n = 56)				1.00
CR	32 (57)	11 (61)	21 (55)	
PR	6 (11)	2 (11)	4 (11)	
SD	4 (7)	1 (6)	3 (8)	
PD	14 (25)	4 (22)	10 (26)	

Bold indicates statistically significant *p*-value

Abbreviations: CNS- central nervous system, yrs-years, COPD-chronic obstructive pulmonary disease, tFL-transformed follicular lymphoma, DLBCL-diffuse large B-cell lymphoma, tMZL-transformed marginal zone lymphoma, IPI-international prognostic index, auto-HCT-autologous hematopoietic cell transplantation, PS-performance status, CR-complete response, PR-partial response, SD-stable disease, PD-progressive disease, Axi-cel- Axicabtagene ciloleucel, Tisa-cel- Tisagenlecleucel, Liso-cel- Lisocabtagene maraleucel, Brexu-cel- Brexucabtagene autoleucel

*Cardiac co-morbidities included H/O coronary artery disease, hypertension, arrhythmias

**Others: PMBL = 1, RT = 1, PTLD = 1, MCL = 1

^Refractory to first-line therapy

At relapse/progression, 20 had CNS only relapse, while 41 had CNS and systemic relapse prior to CAR-T. Median lines of therapy prior to CAR-T were 3 (range, 1–5). The most frequently used CAR-T product was axicabtagene ciloleucel (axi-cel, n = 30, 49%) followed by tisagenlecleucel (tisa-cel, n = 19, 31%) and lisocabtagene maraleucel (liso-cel, n = 11, 18%). Compared to patients with CNS only relapse, those with CNS and systemic relapse were more likely to have DHL (*p* = 0.003), no prior auto-HCT (*p* = 0.03), ECOG performance status of ≥ 2 at apheresis (*p* = 0.02), leptomeningeal involvement (*p* = 0.04), shorter median time from diagnosis to CAR-T (*p* = 0.04), and chemorefractory status prior to CAR-T (< 0.001).

The overall response rate (ORR) was 68% with a CR rate of 57% in the current study. The ORR and CR rates were comparable (*p* = 1.00) between those with CNS only relapse (72%/61%) and CNS and systemic relapse (66%/55%) prior to CAR-T.

Any grade CRS was 70% (n = 43) with 7 patients (16%) experiencing grade 3 or higher CRS. Any grade ICANS was 57% (n = 34) with 15 patients (44%) experiencing grade 3 or higher ICANS. There was no difference in the rate of CRS or ICANS in patients with CNS only versus CNS and systemic relapse prior to CAR-T (Additional file 1: Table S2).

Additional file 1: Table S3 outlines the various factors that were evaluated to determine the association of achievement of complete response (CR vs. no CR) to CAR-T. The majority of the patients in both groups (72% [n = 23] in the CR group and 54% [n = 13] in the no CR group) were chemorefractory prior to CAR-T. Patients who achieved CR to CAR-T were more likely to have had any grade CRS (66% vs. 34%, *p* = 0.037) compared to those who did not achieve CR. In the multivariable analysis, only patients who experienced any grade CRS (compared to no CRS) had higher odds of achieving CR (OR = 3.9, 95% CI = 1.01–15.39, *p* = 0.047), while those who received ≥ 4 lines of therapy (compared to < 4 lines of therapy) trended towards statistical significance for achieving CR (OR = 10.2, 95% CI = 1.00–103.44, p = 0.05).

The median PFS was 3.3 months (95% CI = 2.6–6.0 months, Fig. 1A). The 6-month and 12-month PFS rate was 35% (95% CI = 0.24–0.50) and 16% (95% CI = 0.08–0.30), respectively. Additional file 1: Table S4 shows the median and 6-month PFS rates among the different subgroups. The PFS rates in the subgroups overall remain in line with the main analysis. There was no difference in median PFS based on the type of CAR-T product (Additional file 1: Figure S1A). Of note, patients with ECOG PS of 0–1 at apheresis had longer median PFS compared to those with ECOG PS of ≥ 2, however, this did not reach statistical significance (3.5 months vs. 1.3 months, respectively, *p* = 0.06, Additional file 1: Figure S2). In the multivariable analysis, we did not identify any factors that were associated with significantly superior PFS.

**Fig. 1:**
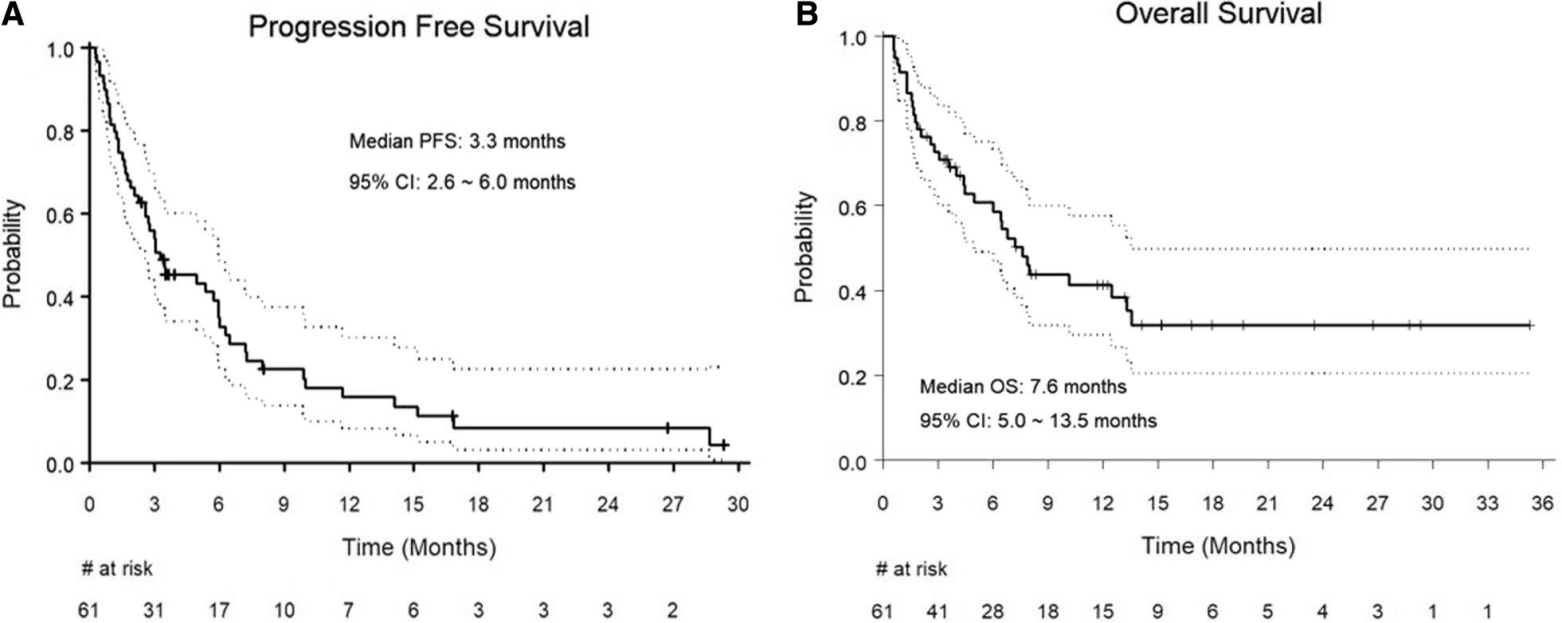
Survival analysis of patients undergoing CAR-T for SCNSL, **A** PFS **B** OS

At a median follow-up time of 14.1 months (95% CI: 11.7–23.5 months), the median OS was 7.6 months (95% CI = 5.0–13.5 months, Fig. 1B). The 6-month and 12-month OS rates were 59% (95% CI = 0.47–0.73) and 41% (95% CI = 0.30–0.57), respectively. Additional file 1: Table S5 shows the median, 6- and 12-month OS rates among the subgroups. The OS rates in the subgroups overall remain in line with the main analysis. There was no difference in median OS based on the type of CAR-T product (Additional file 1: Figure S1B). Of note, patients with no cardiac co-morbidities (7.2 months vs. 4.7 months, *p* = 0.049, Additional file 1: Figure S3A) and ECOG PS of 0–1 at apheresis (12.5 months vs. 2.8 months, Additional file 1: Figure S3B) had significantly longer median OS compared to those with cardiac co-morbidities and ECOG PS of ≥ 2, respectively. While patients with CNS only relapse (NR vs. 6.4 months) had longer median OS compared to those with CNS and systemic relapse, this did not reach statistical significance (*p* = 0.07, Additional file 1: Figure S4). In the multivariable analysis, only ECOG PS of 0–1 at the time of apheresis remained significantly associated with superior OS (HR = 2.56, 95% CI = 1.23–5.32, *p* = 0.01).

The variables evaluated to determine the risk of experiencing CRS (vs. no CRS) to CAR-T are outlined in Additional file 1: Table S6. Only the CAR-T product was significantly different between the two groups (*p* = 0.02). Among the patients who received axi-cel (n = 30), 87% (n = 26) experienced CRS, while 13% (n = 4) did not. In contrast, of patients who received tisa-cel (n = 19), 47% (n = 9) experienced CRS compared to 53% (n = 10) who did not. Additional file 1: Table S7 outlines the variables considered to determine the risk of experiencing NT (vs. no NT) to CAR-T. Only the site of CNS involvement was significantly different between the two groups. Patients who experienced NT had a significantly higher leptomeningeal (61% [n = 17] vs. 39% [n = 11]) and leptomeningeal and parenchymal involvement (100% [n = 6] vs. 0% [n = 0]) compared to those who did not experience NT (*p* = 0.03). While the type of CAR-T product did not reach statistical significance for the risk of NT between the two groups (p = 0.09), a similar trend that was observed with CRS in regard to the CAR-T product was observed with NT. For instance, among the patients who received axi-cel (n = 30), 70% (n = 21) experienced some grade of NT. In contrast, patients who received tisa-cel (n = 19) and liso-cel (n = 10), 42% (n = 8) and 40% (n = 4) experienced NT, respectively.

In this multicenter retrospective cohort study, we evaluated the outcomes of patients with SCNSL who received CAR-T and made several important observations. First, the ORR to CAR-T was high (68%) with the majority of these responses being CR (57%). Second, patients who experienced any grade CRS had a significantly higher probability of achieving CR to CAR-T but did not correlate with superior PFS. Third, the rate of grade ≥ 3 CRS was 16% in line with patients without CNS involvement but with high rates of grade ≥ 3 NT (44%). Fourth, factors associated with increased risk of CRS and NT included the type of CAR-T product and location of CNS involvement, respectively. Lastly, despite the high response rates, the responses were not durable with poor PFS and OS demonstrating the ongoing challenge of treating this patient population.

The ORR to CAR-T in SCNSL patients was in line with what has been reported in the literature, however, the CR rate was higher in our study (57%) compared to the other SNCL studies (which was around 40%) [10, 11]. One potential reason for this may be related to the higher proportion of patients receiving radiation (± chemotherapy) as a bridging therapy in our study. In addition, differences in the CAR-T product could have also contributed to this discrepancy. For instance, in the current study, 49% received axi-cel, 31% received tisa-cel, and 18% liso-cel, which is in contrast to the other studies that were predominated by one CAR-T product (axi-cel in Bennani et al [10] and tisa-cel in Karschnia et al [11]). We found that patients who experienced any grade CRS had a significantly higher probability of achieving CR to CAR-T.

Despite the high CR rates, the patients experienced early therapy failure (short PFS). Furthermore, we did not identify any factors that were associated with significantly longer PFS in the current study. While the exact mechanism for the lack of durable responses is unclear, one potential explanation for this could be the relatively rapid waning of the CAR-T cells in the CNS. However, this hypothesis needs to be tested formally in a prospective manner. One potential strategy to mitigate the early disease progression would be to use maintenance therapy (in those achieving a response) to maintain the remission.

We found the rate of any grade CRS and grade ≥ 3 CRS was comparable to the CAR-T studies in systemic LBCL [5–7] with the highest rate noted with axi-cel. In contrast to systemic LBCL, the grade ≥ 3 ICANS rate was relatively higher than what has been reported in the systemic LBCL and other SCNSL studies [11]. We found that leptomeningeal involvement (with or without parenchymal involvement) portended a higher rate of ICANS, which might be related to disruption of the blood–brain barrier.

The study is subjected to the inherent limitations of a retrospective study design including the non-uniform selection of bridging therapy and CAR-T product. Additionally, we did not collect information on the use of steroids at the time of lymphodepleting chemotherapy or CAR-T, which has been shown to influence outcomes [11, 12]. Lastly, we acknowledge that the numbers in the subgroups were small precluding our ability to identify a population of SCNSL that may have durable remission with CAR-T.

In conclusion, this is the largest study to date, to report outcomes in patients with SCNSL treated with CAR-T and provides a benchmark for studies in SCNSL exploring cellular therapy options in the future. We show that despite achieving high CR rates following CAR-T, these responses are not durable with poor survival underscoring the unmet need for treatment of relapsed and refractory SCNSL. Studies are urgently needed to explore strategies to improve outcomes following CAR-T in SCNSL patients.

### Supplementary Information

**Additional file 1**. Supplementary figures and tables.

## Data Availability

Please email the corresponding author(s).
