# Supplementary material for: Outcomes of patients with secondary central nervous system lymphoma following CAR T-cell therapy: a multicenter cohort study
Source: J Hematol Oncol. 2023 Nov 9;16:111. doi: 10.1186/s13045-023-01508-3 (PMC10633964; doi:10.1186/s13045-023-01508-3)

**SUPPLEMENTAL APPENDIX**

**Table of Contents** 1

**Methods**  2

**CAR T-cell therapy for SCNSL:** Table S1 3-4

**Toxicity post CAR-T:** Table S2 5

**Patient characteristics based on best response of CR post CAR-T:** Table S3 6-7

**PFS of patients receiving CAR-T for SCNSL among subgroups:** Table S4 8

**OS of patients receiving CAR-T for SCNSL among subgroups:** Table S5 9

**Factors associated with risk of CRS:** Table S6 10-11

**Factors associated with risk of Neurotoxicity:** Table S7 12-13

**PFS and OS based on the type of CAR-T product:** Figure S1 14

**PFS of patients receiving CAR-T for SCNSL based on ECOG at apheresis:** Figure S2 15

**OS of patients receiving CAR-T for SCNSL among subgroups:** Figure S3 16

**OS of patients receiving CAR-T for SCNSL among CNS vs CNS and systemic relapse:** Figure S4 17

**Methods**

**Assessment of Toxicity and Response**

CRS and Immune Effector Cell Associated Neurotoxicity Syndrome (ICANS) were graded according to the consensus grading system proposed by the American Society for Transplantation and Cellular Therapy^27^. CNS disease and response to therapy were evaluated according to the local institutional guidelines. Response to CAR-T was graded as CR, partial response, stable disease, or progressive disease.

**Statistical analysis**

Patient demographics, disease characteristics, and clinical outcomes were summarized through descriptive statistics. The Chi-square test or Fisher’s exact test was used to evaluate the association between two categorical variables. Wilcoxon rank sum test was used to evaluate the difference in a continuous variable between patient groups. A multivariable logistic regression model was fitted to estimate the effects of important covariates on best CR post CAR-T. Kaplan-Meier method was used for time-to-event analysis including PFS and OS. Median time to event in months with a 95% confidence interval was calculated. The Log-rank test was used to evaluate the difference in time-to-event endpoints between patient groups. Cox proportional hazard models were used for multivariable analysis. The Schoenfeld residual was used to check the proportional hazards assumption. The variables that had a p-value less than 0.2 for PFS or OS from the univariate analysis were included in the initial full multivariable model. A backward selection method was used and a significant level of 0.10 was set as the criterion for a variable to stay in the model. Collinearity diagnostics were performed and indicated no collinearity problem. Statistical software SAS 9.4 (SAS, Cary, NC) and S-Plus 8.2 (TIBCO Software Inc., Palo Alto, CA) were used for all the analyses.

**Table S1. CAR T-cell therapy for SCNSL (studies ≥5 patients)**

| **Study** | **Study Design** | **CAR-T product** | **Patients (n)** | **Median age at CAR-T, years (range)** |
| --- | --- | --- | --- | --- |
| Frigault ^1^ | Single-center retrospective study | Tisa-cel | 8 | 50 (17-79) |
| Bennani ^2^ | Multicenter retrospective cohort | Axi-cel | 17 | 58 (48-69) |
| Ahmed ^3^ | Single-center retrospective study | Tisa-cel (n=4)  Axi-cel (n=3) | 7 | 50 (39-72) |
| Ghafouri^4^ | Single-center retrospective study | Axi-cel | 5 | 58 (28-76) |
| Karschnia^5^ | Single-center retrospective study | Tisa-cel (n=20)  Liso-cel (n=7) | 27 | 33^ |
| Zurko^6^ | Multi-center retrospective study | NR^^ | 32^^^^ | 59^^^^ |
| Abramson^7^ | clinical trial  TRANSCEND | Liso-cel | 7 | 63 (54-70)* |
| Wu^8^ | clinical trial  prospective study | Experimental** | 9 | 47 (23-65) |
| Liu^9^ | clinical trial  prospective study | CD19 or CD20 CAR T-cell therapy | 6 | 43 (43-66) |

Abbreviations: Tisa-cel: Tisagenlecleucel; Axi-cel: Axicabtagene Ciloleucel; Liso-cel: [Lisocabtagene maraleucel](https://www.sciencedirect.com/topics/medicine-and-dentistry/lisocabtagene-maraleucel); NR: not reported

^only provided the mean and SD (32.7± 1.4) years

^^^^The details on the CNSL (including relapsed primary versus secondary CNSL) and CAR-T product for the CNSL cohort were not provided. The age is for the entire group and not specifically for the CNSL patients

*This is for the entire group and not specifically for the SCNSL patients.

**CD19/CD22 CAR T-cell therapy

**References**

1. Frigault MJ, Dietrich J, Martinez-Lage M, et al. Tisagenlecleucel CAR T-cell therapy in secondary CNS lymphoma. *Blood*. 2019;134(11):860-866.

2. Bennani NN MM, Nastoupil LJ, Jain MD, Chavez JC, Cashen AF, et al. Experience with Axicabtagene Ciloleucel (Axi-cel) in Patients with Secondary CNS Involvement: Results from the US Lymphoma CAR T Consortium. ***Blood***. 2019;134:763.

3. Ahmed G, Hamadani M, Shah NN. CAR T-cell therapy for secondary CNS DLBCL. *Blood Adv*. 2021;5(24):5626-5630.

4. Ghafouri S, Timmerman J, Larson S, Mead MD. Axicabtagene Ciloleucel CAR T-cell therapy for relapsed/refractory secondary CNS non-Hodgkin lymphoma: comparable outcomes and toxicities, but shorter remissions may warrant alternative consolidative strategies? *Bone Marrow Transplantation*. 2021;56(4):974-977.

5. Karschnia P, Rejeski K, Winkelmann M, et al. Toxicities and Response Rates of Secondary CNS Lymphoma After Adoptive Immunotherapy With CD19-Directed Chimeric Antigen Receptor T Cells. *Neurology*. 2022;98(21):884-889.

6. Zurko J, Nizamuddin I, Epperla N, et al. Peri-CAR-T practice patterns and survival predictors for all CAR-T patients and post-CAR-T failure in aggressive B-NHL. *Blood Adv*. 2023;7(12):2657-2669.

7. Abramson JS, Palomba ML, Gordon LI, et al. Lisocabtagene maraleucel for patients with relapsed or refractory large B-cell lymphomas (TRANSCEND NHL 001): a multicentre seamless design study. *Lancet*. 2020;396(10254):839-852.

8. Wu J, Meng F, Cao Y, et al. Sequential CD19/22 CAR T-cell immunotherapy following autologous stem cell transplantation for central nervous system lymphoma. *Blood Cancer J*. 2021;11(7):131.

9. CD19 or CD20 CAR T Cell Therapy Demonstrates Durable Antitumor Efficacy in Patients with Central Nervous System Lymphoma. *Human Gene Therapy*. 2022;33(5-6):318-329.

**Table S2. Toxicity post CAR-T**

| **Variable** | **All patients**  **N=61 (%)** | **CNS only**  **N=20 (%)** | **CNS and systemic**  **N=41 (%)** |
| --- | --- | --- | --- |
| Any grade CRS | 43 (70) | 15 (75) | 28 (68) |
| Grade ≥ 3 CRS* | 7 (16) | 3 (15) | 4 (10) |
| Any grade ICANS | 34 (57) | 13 (65) | 21 (52) |
| Grade ≥ 3 ICANS** | 15 (44) | 3 (15) | 12 (30) |
| Steroids | 32 (59) | 11 (69) | 21 (55) |
| Tocilizumab | 28 (47) | 10 (53) | 18 (44) |

Abbreviations: CRS-cytokine release syndrome. ICANS- immune effector cell-associated neurotoxicity syndrome

*Among the patients who had CRS

** Among the patients who had ICANS

**Table S3. Patient characteristics based on the best response of CR post CAR-T**

| **Variable** | **All patients**  **N=56 (%)** | **CR**  **N=32 (%)** | **No CR**  **N=24 (%)** | **p-value** |
| --- | --- | --- | --- | --- |
| Age at diagnosis |  |  |  | 0.33 |
| Median, range (yrs) | 56 (18-73) | 56 (18-70) | 56 (23-73) |  |
| >60 yrs | 16 (29) | 7 (44) | 9 (56) | 0.20 |
| ≤60 yrs | 40 (71) | 25 (62) | 15 (37) |  |
| Sex, n (%) |  |  |  | 0.70 |
| Males | 31 (55) | 17 (53) | 14 (58) |  |
| Females | 25 (45) | 15 (47) | 10 (42) |  |
| IPI at diagnosis |  |  |  | 0.27 |
| 0 | 1 (2) | 1 (4) | 0 |  |
| 1-2 | 14 (29) | 6 (21) | 8 (40) |  |
| ≥3 | 33 (69) | 21 (75) | 12 (60) |  |
| DHL | 15 (32) | 8 (31) | 7 (33) | 0.58 |
| Primary refractory disease |  |  |  | 0.10 |
| Yes | 12 (22) | 4 (13) | 8 (33) |  |
| No | 43 (78) | 27 (87) | 16 (67) |  |
| Lines of therapy prior to CAR-T |  |  |  | 0.04 |
| ≤2 | 18 (33) | 9 (29) | 9 (39) |  |
| 3 | 25 (46) | 12 (39) | 13 (57) |  |
| ≥4 | 11 (20) | 10 (32) | 1 (4) |  |
| Prior auto-HCT |  |  |  | 0.52 |
| Yes | 12 (21) | 8 (25) | 4 (17) |  |
| No | 44 (79) | 24 (75) | 20 (83) |  |
| Median time from diagnosis to CAR-T, range (months) | 15.8 (3.6–257.4) | 16.8 (7.3-257.4) | 15.6 (3.6-130-3) | 0.61 |
| CAR-T product |  |  |  | 0.76 |
| Axi-cel | 27 (48) | 16 (50) | 11 (46) |  |
| Tisa-cel | 17 (30) | 8 (25) | 9 (37) |  |
| Liso-cel | 11 (20) | 7 (22) | 4 (17) |  |
| Brexu-cel | 1 (2) | 1 (3) | 0 |  |
| Median age at apheresis, range (yrs) | 58 (18-74) | 58 (18-72) | 57 (23-74) | 0.35 |
| ECOG PS at apheresis |  |  |  | 0.32 |
| 0-1 | 44 (79) | 27 (84) | 17 (71) |  |
| ≥2 | 12 (21) | 5 (16) | 7 (29) |  |
| Type of relapse |  |  |  | 0.68 |
| CNS only | 18 (32) | 11 (34) | 7 (29) |  |
| CNS + systemic | 38 (68) | 21 (66) | 17 (71) |  |
| Site of CNS involvement |  |  |  | 0.43 |
| Parenchymal | 23 (42) | 12 (39) | 11 (46) |  |
| Leptomeningeal | 26 (47) | 14 (45) | 12 (50) |  |
| Both | 6 (11) | 5 (16) | 1 (4) |  |
| Disease status prior to CAR-T |  |  |  | 0.34 |
| CR | 3 (5) | 2 (6) | 1 (4) |  |
| PR | 17 (30) | 7 (22) | 10 (42) |  |
| SD | 9 (16) | 7 (22) | 2 (8) |  |
| PD | 27 (48) | 16 (50) | 11 (46) |  |
| Bridging |  |  |  | 0.41 |
| Chemo only | 20 (36) | 9 (28) | 11 (46) |  |
| Chemo+XRT | 5 (9) | 3 (9) | 2 (8) |  |
| XRT only | 9 (16) | 7 (22) | 2 (8) |  |
| None | 22 (39) | 13 (41) | 9 (38) |  |
| Any grade CRS |  |  |  | **0.037** |
| Yes | 41 (73) | 27 (84) | 14 (58) |  |
| No | 15 (27) | 5 (16) | 10 (42) |  |
| Any grade ICANS |  |  |  | 0.40 |
| Yes | 31 (56) | 19 (61) | 12 (50) |  |
| No | 24 (44) | 12 (39) | 12 (50) |  |

Abbreviations: CNS- central nervous system, yrs-years, IPI-international prognostic index, auto-HCT-autologous hematopoietic cell transplantation, PS-performance status, CR-complete response, PR-partial response, SD-stable disease, PD-progressive disease, CRS-cytokine release syndrome. ICANS- immune effector cell-associated neurotoxicity syndrome, Axi-cel- Axicabtagene ciloleucel, Tisa-cel- Tisagenlecleucel, Liso-cel- Lisocabtagene maraleucel, Brexu-cel- Brexucabtagene autoleucel

**Table S4. PFS of patients receiving CAR-T for SCNSL among the various subgroups**

| **Variable** | **Level** | **Median PFS time in mos (95%CI)** | **PFS rate at 6 mos (95%CI)** | **p-value** |
| --- | --- | --- | --- | --- |
| Age | <=60 | 3.48 (2.56 , 7.26 ) | 0.4 (0.27 , 0.59 ) | 0.51 |
|  | >60 | 3.02 (1.61 , 6.01 ) | 0.24 (0.1 , 0.55 ) |  |
| Sex | Female | 5.35 (3.02 , 7.19 ) | 0.41 (0.25 , 0.66 ) | 0.71 |
|  | Male | 2.64 (1.61 , 7.98 ) | 0.3 (0.17 , 0.52 ) |  |
| COO | GC | 5.98 (2.73 , 14.13 ) | 0.47 (0.3 , 0.72 ) | 0.17 |
|  | Non-GCB/ ABC | 2.56 (1.51 , 6.01 ) | 0.27 (0.14 , 0.52 ) |  |
| DHL | Yes | 2.74 (1.25 , 7.26 ) | 0.19 (0.07 , 0.52 ) | 0.60 |
| IPI at diagnosis | 0 | NA (NA , NA ) |  | 0.50 |
|  | 1 | 1.71 (0.46 , NA ) | 0.25 (0.05 , 1 ) |  |
|  | 2 | 2.33 (1.25 , NA ) | 0.3 (0.12 , 0.77 ) |  |
|  | 3 | 4.96 (2.56 , 7.26 ) | 0.26 (0.12 , 0.59 ) |  |
|  | 4 | 3.48 (1.74 , 15.18 ) | 0.41 (0.23 , 0.75 ) |  |
| COPD | No | 3.02 (2.27 , 5.95 ) | 0.31 (0.21 , 0.47 ) | 0.37 |
|  | Yes | 7.26 (6.27 , NA ) | 1 (1 , 1 ) |  |
| Cardiac comorbidities | No | 3.45 (2.56 , 7.19 ) | 0.38 (0.26 , 0.56 ) | 0.15 |
|  | Yes | 2.18 (0.95 , NA ) | 0.25 (0.09 , 0.67 ) |  |
| Prior auto-HCT | No | 2.99 (1.74 , 6.01 ) | 0.34 (0.23 , 0.52 ) | 0.33 |
|  | Yes | 4.42 (3.02 , NA ) | 0.37 (0.17 , 0.83 ) |  |
| ECOG at apheresis | ≥2 | 1.3 (0.82 , NA ) | 0.18 (0.05 , 0.6 ) | 0.06 |
|  | 0-1 | 3.48 (2.76 , 7.26 ) | 0.4 (0.28 , 0.58 ) |  |
| Type of relapse | CNS only | 6.01 (2.07 , 11.7 ) | 0.55 (0.36 , 0.84 ) | 0.34 |
|  | CNS and systemic | 2.99 (2.27 , 5.95 ) | 0.27 (0.16 , 0.45 ) |  |
| Site of CNS involvement | Parenchymal | 3.02 (2.27 , 6.27 ) | 0.3 (0.16 , 0.56 ) | 0.47 |
|  | Leptomeningeal | 2.76 (1.68 , 7.98 ) | 0.37 (0.22 , 0.61 ) |  |
|  | Both | 5.95 (5.75 , NA ) | 0.4 (0.14 , 1 ) |  |
| Bridging | Chemo only | 1.63 (1.15 , 5.75 ) | 0.13 (0.04 , 0.44 ) | 0.23 |
|  | Chemo/XRT | 5.35 (2.99 , NA ) | 0.3 (0.06 , 1 ) |  |
|  | XRT only | 5.95 (1.61 , NA ) | 0.44 (0.21 , 0.92 ) |  |
|  | None | 6.01 (3.02 , 11.7 ) | 0.5 (0.33 , 0.76 ) |  |
| CAR-T product | Axi-cel | 5.35 (2.56 , 7.26 ) | 0.36 (0.21 , 0.59 ) | 0.95 |
|  | Tisa-cel | 2.99 (1.68 , 9.99 ) | 0.32 (0.16 , 0.61 ) |  |
|  | Liso-cel | 4.96 (2.27 , NA ) | 0.35 (0.13 , 0.95 ) |  |

**Table S5: Overall survival of patients receiving CAR-T for SCNSL among the various subgroups**

| **Variable** | **Level** | **Median OS time in mos (95%CI)** | **OS rate at 6 mos (95%CI)** | **OS rate at 1 year (95%CI)** | **p-value** |
| --- | --- | --- | --- | --- | --- |
| Age | <=60 | 7.59 (5.98 , NA ) | 0.61 (0.47 , 0.79 ) | 0.42 (0.28 , 0.63 ) | 0.73 |
|  | >60 | 6.44 (2.07 , NA ) | 0.52 (0.33 , 0.83 ) | 0.39 (0.21 , 0.72 ) |  |
| Sex | Female | 7.59 (6.44 , NA ) | 0.69 (0.53 , 0.89 ) | 0.45 (0.29 , 0.71 ) | 0.33 |
|  | Male | 5.98 (4.37 , NA ) | 0.5 (0.35 , 0.72 ) | 0.38 (0.23 , 0.61 ) |  |
| COO | GC | 10.15 (6.44 , NA ) | 0.71 (0.54 , 0.92 ) | 0.49 (0.32 , 0.77 ) | 0.23 |
|  | Non-GCB/ ABC | 6.77 (2.79 , NA ) | 0.55 (0.39 , 0.79 ) | 0.35 (0.2 , 0.62 ) |  |
| DHL | Yes | 4.47 (3.58 , NA ) | 0.34 (0.17 , 0.69 ) | 0.2 (0.07 , 0.56 ) | 0.41 |
| IPI at diagnosis | 0 | NA (NA , NA ) |  |  | 0.77 |
|  | 1 | 7.51 (0.89 , NA ) | 0.5 (0.19 , 1 ) |  |  |
|  | 2 | 6.47 (2.79 , NA ) | 0.56 (0.31 , 1 ) | 0.28 (0.09 , 0.88 ) |  |
|  | 3 | 6.77 (4.96 , NA ) | 0.61 (0.42 , 0.89 ) | 0.36 (0.19 , 0.69 ) |  |
|  | 4 | 7.98 (3.58 , NA ) | 0.59 (0.38 , 0.91 ) | 0.44 (0.25 , 0.8 ) |  |
| COPD | No | 6.77 (4.47 , 13.3 ) | 0.56 (0.44 , 0.72 ) | 0.38 (0.26 , 0.55 ) | 0.09 |
|  | Yes | NA (NA , NA ) | 1 (1 , 1 ) | 1 (1 , 1 ) |  |
| Cardiac comorbidities | No | 7.59 (6.44 , NA ) | 0.64 (0.51 , 0.8 ) | 0.5 (0.36 , 0.68 ) | **0.049** |
|  | Yes | 4.71 (1.61 , NA ) | 0.42 (0.21 , 0.81 ) | 0.12 (0.02 , 0.68 ) |  |
| Prior auto-HCT | No | 7.16 (4.47 , NA ) | 0.56 (0.43 , 0.72 ) | 0.4 (0.27 , 0.58 ) | 0.56 |
|  | Yes | 7.59 (4.37 , NA ) | 0.7 (0.46 , 1 ) | 0.47 (0.23 , 0.94 ) |  |
| ECOG at apheresis | ≥2 | 2.79 (1.58 , NA ) | 0.4 (0.21 , 0.78 ) | 0.1 (0.02 , 0.62 ) | **0.005** |
|  | 0-1 | 12.45 (6.44 , NA ) | 0.64 (0.51 , 0.81 ) | 0.5 (0.37 , 0.69 ) |  |
| Type of relapse | CNS only | NA (7.98 , NA ) | 0.77 (0.6 , 1 ) | 0.63 (0.43 , 0.92 ) | 0.07 |
|  | CNS and systemic | 6.44 (4.01 , 13.3 ) | 0.5 (0.37 , 0.69 ) | 0.32 (0.19 , 0.52 ) |  |
| Site of CNS involvement | Parenchymal | 7.85 (5.98 , NA ) | 0.66 (0.49 , 0.89 ) | 0.41 (0.24 , 0.68 ) | 0.39 |
|  | Leptomeningeal | 4.96 (3.02 , NA ) | 0.46 (0.3 , 0.7 ) | 0.31 (0.17 , 0.58 ) |  |
|  | Both | NA (13.53 , NA ) | 0.8 (0.52 , 1 ) | 0.8 (0.52 , 1 ) |  |
| Bridging | Chemo only | 3.63 (1.68 , NA ) | 0.38 (0.21 , 0.67 ) | 0.23 (0.1 , 0.57 ) | 0.20 |
|  | Chemo/XRT | 6.77 (5.98 , NA ) | 0.53 (0.21 , 1 ) | 0.27 (0.05 , 1 ) |  |
|  | XRT only | 13.53 (6.47 , NA ) | 0.76 (0.52 , 1 ) | 0.51 (0.26 , 1 ) |  |
|  | None | 12.45 (6.44 , NA ) | 0.72 (0.55 , 0.94 ) | 0.55 (0.37 , 0.83 ) |  |
| CAR-T product | Axi-cel | 7.98 (4.47 , NA ) | 0.64 (0.48 , 0.85 ) | 0.42 (0.26 , 0.67 ) | 0.93 |
|  | Tisa-cel | 4.37 (2.56 , NA ) | 0.41 (0.23 , 0.71 ) | 0.41 (0.23 , 0.71 ) |  |
|  | Liso-cel | 7.85 (6.44 , NA ) | 0.78 (0.55 , 1 ) | 0.39 (0.16 , 0.94 ) |  |

**Table S6. Factors associated with the risk of CRS**

| **Variable** | **All patients**  **N=61 (%)** | **CRS**  **N=43 (%)** | **No CRS**  **N=18 (%)** | **p-value** |
| --- | --- | --- | --- | --- |
| Age at diagnosis |  |  |  | 0.54 |
| Median, range (yrs) | 56 (18-82) | 55 (18-82) | 59 (29-71) |  |
| >60 yrs | 17 (28) | 11 (26) | 6 (33) |  |
| ≤60 yrs | 44 (72) | 32 (74) | 12 (67) |  |
| Sex, n (%) |  |  |  | 0.27 |
| Males | 34 (56) | 22 (51) | 12 (67) |  |
| Females | 27 (44) | 21 (49) | 6 (33) |  |
| IPI at diagnosis |  |  |  | 0.38 |
| 0 | 1 (2) | 1 (3) | 0 |  |
| 1-2 | 15 (29) | 9 (24) | 6 (46) |  |
| ≥3 | 35 (69) | 28 (74) | 7 (54) |  |
| DHL | 16 (31) | 10 (28) | 6 (37) | 0.56 |
| Primary refractory disease |  |  |  | 0.42 |
| Yes | 15 (25) | 10 (23) | 5 (29) |  |
| No | 45 (75) | 33 (67) | 12 (71) |  |
| Median lines of therapy prior to CAR-T, range | 3 (1-5) | 3 (1-5) | 3 (1-4) | 0.41 |
| Prior auto-HCT |  |  |  | 0.74 |
| Yes | 14 (23) | 9 (21) | 5 (28) |  |
| No | 47 (77) | 34 (79) | 13 (72) |  |
| CAR-T product |  |  |  | **0.016** |
| Axi-cel | 30 (49) | 26 (61) | 4 (22) |  |
| Tisa-cel | 19 (31) | 9 (21) | 10 (56) |  |
| Liso-cel | 11 (18) | 7 (16) | 4 (22) |  |
| Brexu-cel | 1 (2) | 1 (2) | 0 |  |
| Median age at apheresis, range (yrs) | 58 (18-83) | 57 (18-83) | 61 (29-71) | 0.34 |
| ECOG PS at apheresis |  |  |  | 1.00 |
| 0-1 | 47 (77) | 33 (77) | 14 (78) |  |
| ≥2 | 14 (23) | 10 (23) | 4 (22) |  |
| Type of relapse |  |  |  | 0.77 |
| CNS only | 20 (33) | 15 (35) | 5 (28) |  |
| CNS + systemic | 41 (67) | 28 (65) | 13 (72) |  |
| Site of CNS involvement |  |  |  | 0.30 |
| Parenchymal | 25 (42) | 17 (41) | 8 (44) |  |
| Leptomeningeal | 29 (48) | 19 (45) | 10 (56) |  |
| Both | 6 (10) | 6 (14) | 0 |  |
| Disease status prior to CAR-T |  |  |  | 0.46 |
| CR | 3 (5) | 3 (7) | 0 |  |
| PR | 19 (31) | 15 (35) | 4 (22) |  |
| SD | 9 (15) | 5 (12) | 4 (22) |  |
| PD | 30 (49) | 20 (46) | 10 (56) |  |
| Bridging |  |  |  | 0.97 |
| Chemo only | 23 (38) | 16 (37) | 7 (39) |  |
| Chemo+XRT | 5 (8) | 4 (9) | 1 (6) |  |
| XRT only | 9 (15) | 7 (16) | 2 (11) |  |
| None | 24 (39) | 16 (37) | 8 (44) |  |

Abbreviations: CRS-cytokine release syndrome, CNS- central nervous system, yrs-years, IPI-international prognostic index, auto-HCT-autologous hematopoietic cell transplantation, PS-performance status, CR-complete response, PR-partial response, SD-stable disease, PD-progressive disease, Axi-cel- Axicabtagene ciloleucel, Tisa-cel- Tisagenlecleucel, Liso-cel- Lisocabtagene maraleucel, Brexu-cel- Brexucabtagene autoleucel

*Among the evaluable patients (n=56)

**Table S7. Factors associated with the risk of Neurotoxicity**

| **Variable** | **All patients**  **N=60 (%)** | **NT**  **N=34 (%)** | **No NT**  **N=26 (%)** | **p-value** |
| --- | --- | --- | --- | --- |
| Age at diagnosis |  |  |  | 0.43 |
| Median, range (yrs) | 56 (18-82) | 56 (23-82) | 56 (18-73) |  |
| >60 yrs | 17 (28) | 11 (32) | 6 (23) |  |
| ≤60 yrs | 43 (72) | 23 (68) | 20 (77) |  |
| Sex, n (%) |  |  |  | 0.71 |
| Males | 33 (55) | 18 (53) | 15 (58) |  |
| Females | 27 (45) | 16 (47) | 11 (42) |  |
| IPI at diagnosis |  |  |  | 0.36 |
| 0 | 1 (2) | 1 (4) | 0 |  |
| 1-2 | 15 (30) | 6 (23) | 9 (37) |  |
| ≥3 | 34 (68) | 19 (73) | 15 (63) |  |
| DHL | 15 (29) | 6 (19) | 9 (45) | 0.12 |
| Primary refractory disease |  |  |  | 0.55 |
| Yes | 15 (25) | 10 (29) | 5 (19) |  |
| No | 45 (75) | 24 (71) | 21 (81) |  |
| Median lines of therapy prior to CAR-T, range | 3 (1-5) | 3 (1-5) | 3 (1-5) | 0.81 |
| Prior auto-HCT |  |  |  | 0.97 |
| Yes | 14 (23) | 8 (23) | 6 (23) |  |
| No | 46 (77) | 26 (77) | 20 (77) |  |
| CAR-T product |  |  |  | 0.09 |
| Axi-cel | 30 (50) | 21 (62) | 9 (35) |  |
| Tisa-cel | 19 (32) | 8 (23) | 11 (42) |  |
| Liso-cel | 10 (17) | 4 (12) | 6 (23) |  |
| Brexu-cel | 1 (2) | 1 (3) | 0 |  |
| Median age at apheresis, range (yrs) | 58 (18-83) | 59 (23-83) | 58 (18-74) | 0.49 |
| ECOG PS at apheresis |  |  |  | 0.56 |
| 0-1 | 46 (77) | 25 (74) | 21 (81) |  |
| ≥2 | 14 (23) | 9 (26) | 5 (19) |  |
| Type of relapse |  |  |  | 0.36 |
| CNS only | 20 (33) | 13 (38) | 7 (27) |  |
| CNS + systemic | 40 (67) | 21 (62) | 19 (73) |  |
| Site of CNS involvement |  |  |  | **0.03** |
| Parenchymal | 25 (42) | 11 (32) | 14 (56) |  |
| Leptomeningeal | 28 (48) | 17 (50) | 11 (44) |  |
| Both | 6 (10) | 6 (18) | 0 |  |
| Disease status prior to CAR-T |  |  |  | 0.32 |
| CR | 3 (5) | 3 (9) | 0 |  |
| PR | 19 (32) | 9 (26) | 10 (38) |  |
| SD | 8 (13) | 6 (18) | 2 (8) |  |
| PD | 30 (50) | 16 (47) | 14 (54) |  |
| Bridging |  |  |  | 0.87 |
| Chemo only | 22 (37) | 13 (38) | 9 (35) |  |
| Chemo+XRT | 5 (8) | 3 (9) | 2 (8) |  |
| XRT only | 9 (15) | 6 (18) | 3 (11) |  |
| None | 24 (40) | 12 (35) | 12 (46) |  |

Abbreviations: NT-neurotoxicity, CNS- central nervous system, yrs-years, IPI-international prognostic index, auto-HCT-autologous hematopoietic cell transplantation, PS-performance status, CR-complete response, PR-partial response, SD-stable disease, PD-progressive disease, Axicabtagene ciloleucel, Tisa-cel- Tisagenlecleucel, Liso-cel- Lisocabtagene maraleucel, Brexu-cel- Brexucabtagene autoleucel

**Figure S1. PFS and OS based the type of CAR-T product**

1. **PFS based on the type of CAR-T product**


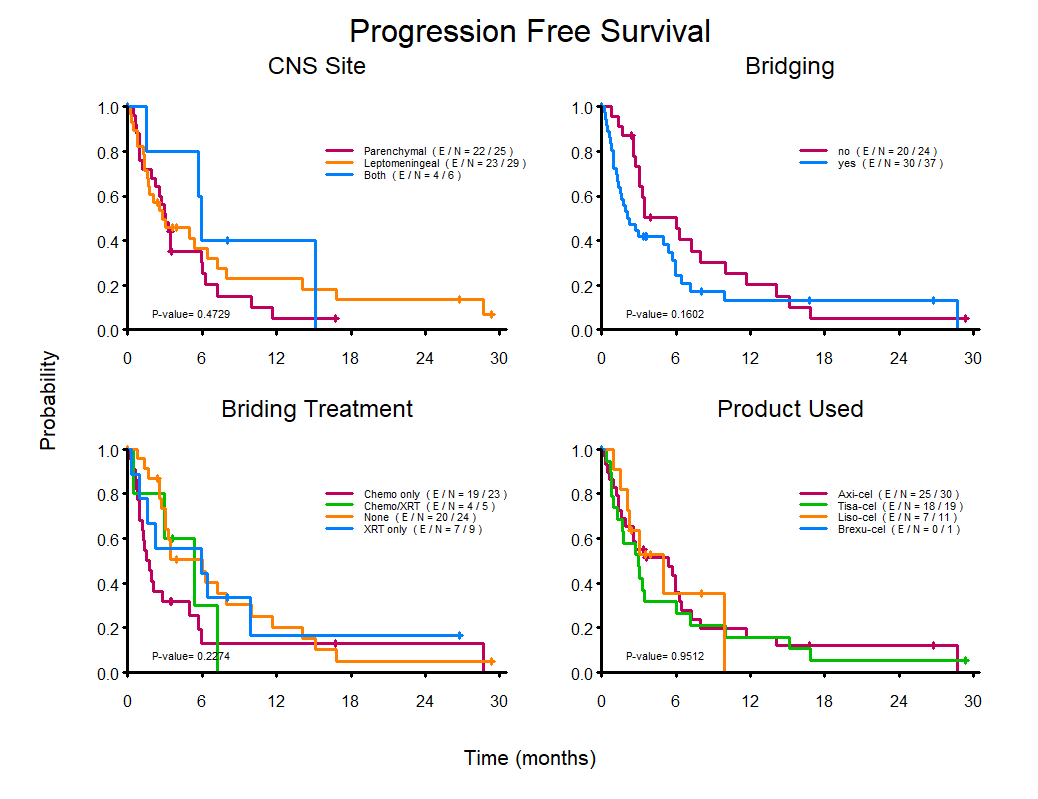


1. **OS based on the type of CAR-T product**


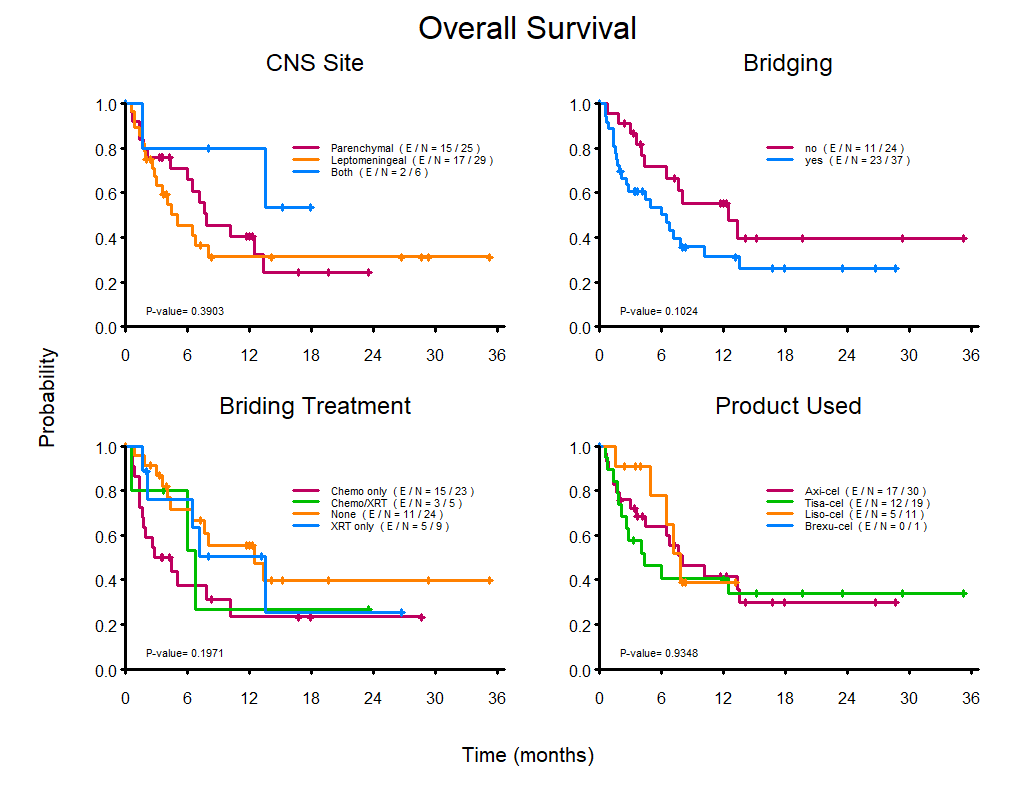


**Figure S2. PFS of patients receiving CAR-T for SCNSL among those with ECOG of 0-1 versus ≥2 at apheresis**


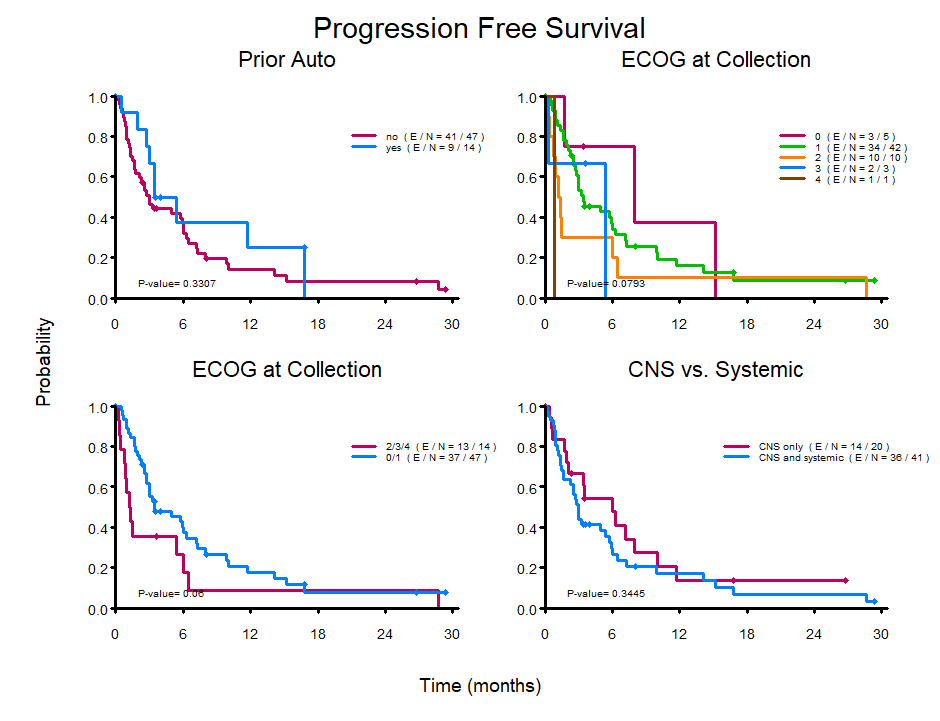


**Figure S3. OS of patients receiving CAR-T for SCNSL among those A) with or without cardiac co-morbidities B) ECOG of 0-1 versus ≥2 at apheresis**

1. **OS based on presence or absence of cardiac co-morbidities**


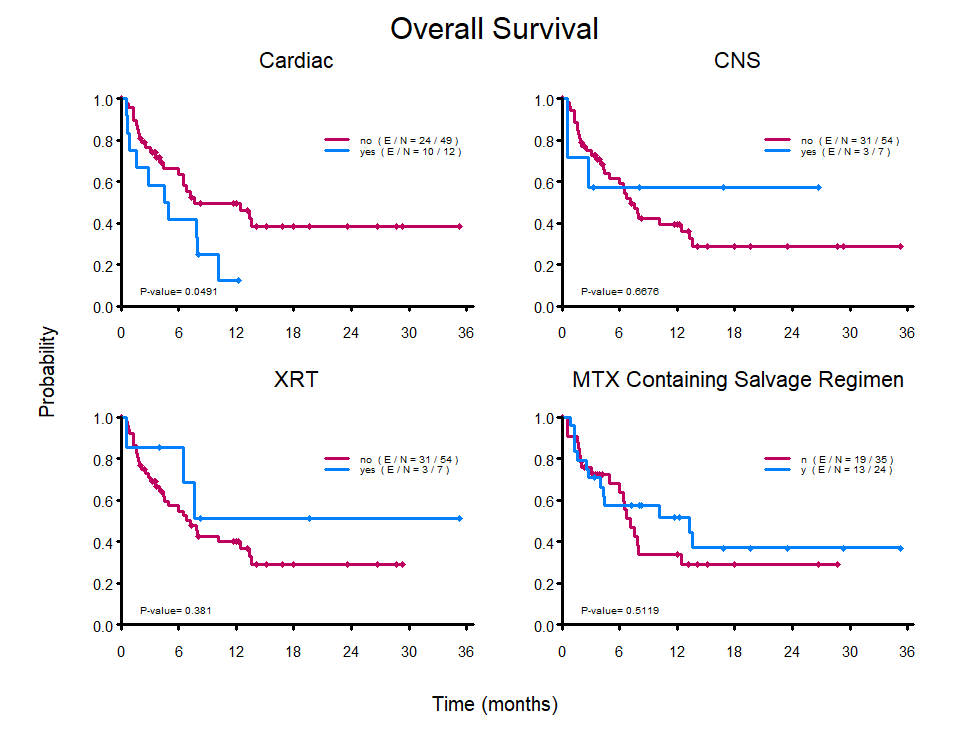


1. **OS based on ECOG of 0-1 versus ≥2 at apheresis**


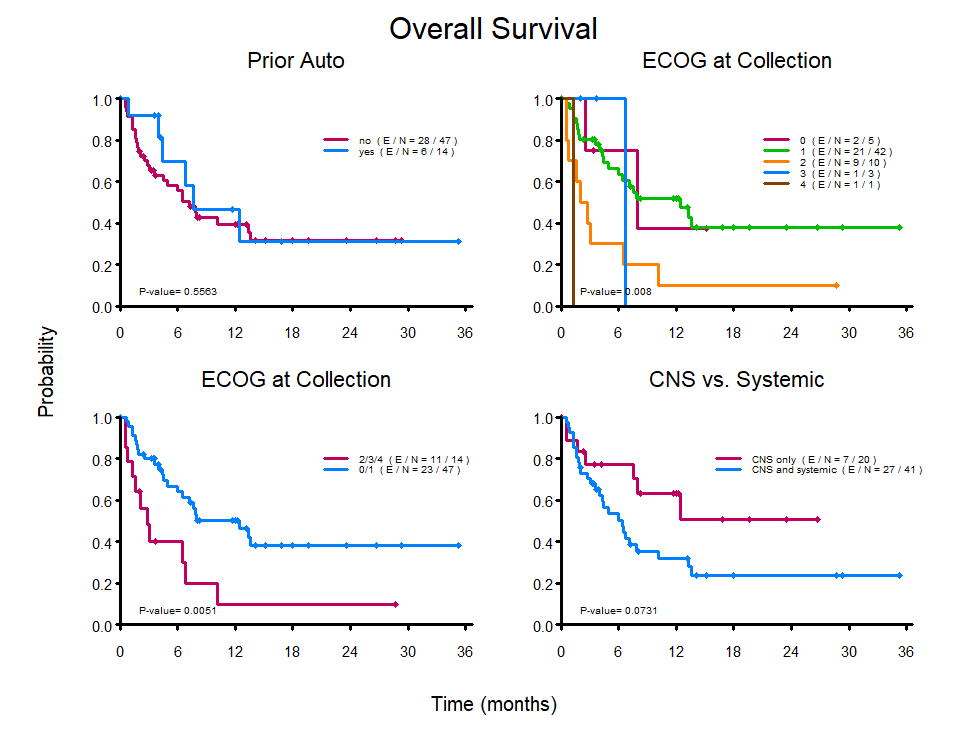


**Figure S4. OS of patients receiving CAR-T for SCNSL among those with CNS only versus CNS and systemic relapse**


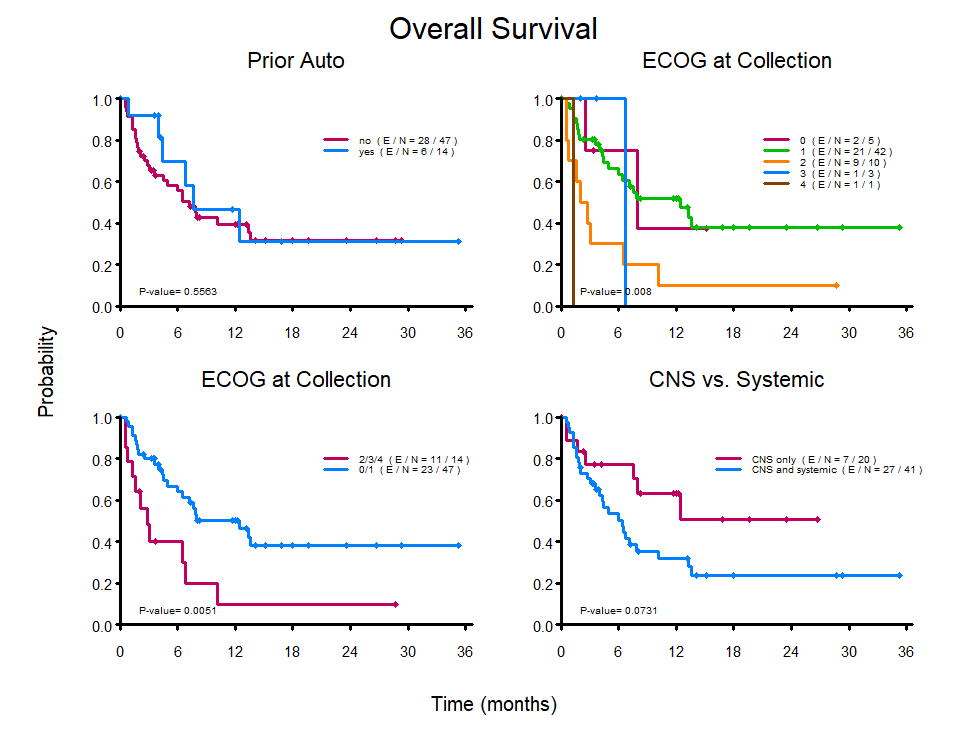

Supplement: Supplementary file 1 — Additional file 1. Supplementary figures and tables. [file 13045_2023_1508_MOESM1_ESM.docx]
